# The inflammatory molecules IL-1β and HMGB1 can rapidly enhance focal seizure generation in a brain slice model of temporal lobe epilepsy

**DOI:** 10.3389/fncel.2014.00155

**Published:** 2014-06-06

**Authors:** Angela Chiavegato, Emanuele Zurolo, Gabriele Losi, Eleonora Aronica, Giorgio Carmignoto

**Affiliations:** ^1^Department of Biomedical Sciences and CNR Institute of Neuroscience, University of PadovaPadova, Italy; ^2^Department of (Neuro)Pathology, Academic Medical Center, and Swammerdam Institute for Life Sciences, Center for Neuroscience, University of Amsterdam, Amsterdam, Netherlands; ^3^SEIN - Stichting Epilepsie Instellingen NederlandHeemstede, Netherlands

**Keywords:** epileptogenesis, seizures, entorhinal cortex, calcium, proinflammatory cytokines, IL-1beta, HMGB1, astrocytes

## Abstract

Epilepsy is a neurological disorder characterized by a hyperexcitable brain tissue and unpredictable seizures, i.e., aberrant firing discharges in large neuronal populations. It is well established that proinflammatory cytokines, in addition to their canonical involvement in the immune response, have a crucial role in the mechanism of seizure generation. The purpose of the present study was to investigate the role of interleukin-1β (IL-1β) and high mobility group B1 (HMGB1) in the generation of seizure-like discharges using two models of focal epilepsy in a rat entorhinal cortex slice preparation. Seizure like-discharges were evoked by either slice perfusion with low Mg^2+^ and picrotoxin or with a double NMDA local stimulation in the presence of the proconvulsant 4-amino-pyridine. The effects of IL-1β or HMGB1 were evaluated by monitoring seizure discharge generation through laser scanning microscope imaging of Ca^2+^ signals from neurons and astrocytes. In the picrotoxin model, we revealed that both cytokines increased the mean frequency of spontaneous ictal-like discharges, whereas only IL-1β reduced the latency and prolonged the duration of the first ictal-like event. In the second model, a single NMDA pulse, *per se* ineffective, became successful when it was performed after IL-β or HMGB1 local applications. These findings demonstrate that both IL-1β and HMGB1 can rapidly lower focal ictal event threshold and strengthen the possibility that targeting these inflammatory pathways may represent an effective therapeutic strategy to prevent seizures.

## Introduction

Epilepsy is a neurological disorder characterized by recurring, unprovoked seizures. The age-adjusted incidence of epilepsy ranges from 16 to 51 per 100,000 worldwide, with higher prevalence in developing countries (Banerjee et al., 2009; Peljto et al., 2014). Increasing evidence supports the involvement of inflammatory and immune processes in the etiopathogenesis of seizures (Vezzani and Granata, 2005; Vezzani et al., 2008, 2010). Inflammation induced by brain-damaging events such as trauma, stroke, infection, hyperthermia, and status epilepticus are associated with acute symptomatic seizures and a high risk of developing epilepsy (Pitkanen and Sutula, 2002; Bartfai et al., 2007). In particular, high levels of proinflammatory cytokines [e.g., interleukin-1beta (IL-1β), tumor necrosis factor-alpha (TNFα)], damage signals [high-mobility group box 1 (HMGB1), S100 beta] and downstream inflammatory mediators (e.g., prostaglandins, the complement system) have been measured in epileptogenic tissue from patients affected by epilepsy of various etiologies (Aronica and Crino, 2011; Vezzani et al., 2011). The major contributors to the synthesis of these inflammatory mediators are brain-resident cells such as activated microglia, astrocytes, and neurons (Devinsky et al., 2013), but also systemic invading leukocytes play an important role in epileptogenesis, particularly when the permeability of the blood-brain barrier is alterated (Fabene et al., 2008; Deprez et al., 2011).

Recently, two proinflammatory molecules were found to be proconvulsant in animal models of temporal lobe epilepsy (TLE): IL-1β and HMGB1 (Vezzani et al., 1999; Ravizza et al., 2008; Maroso et al., 2011). These molecules, applied *in vivo* before the induction of experimental TLE, were able to increase the time spent in seizures and reduce the onset time of the first seizure. The effects of HMGB1 and IL-1β are blocked by ifenprodil (Balosso et al., 2008; Maroso et al., 2010), a selective antagonist of NR2B-containing NMDA receptors (Yu et al., 1997).

Previous studies also reported that astrocytes can promote episodes of synchronous activity in the neuronal network (Fellin et al., 2004) and that this action may contribute to ictal discharge generation (Gomez-Gonzalo et al., 2010; Losi et al., 2010). Using two different focal seizure models in cortical slice preparations we investigate whether two pro-inflammatory cytokines, i.e., IL-1β and HMGB1, can affect neuronal excitability and favor the generation of epileptic activity. In the first model, slices from the entorhinal cortex (EC) were perfused with picrotoxin in the virtual absence of extracellular Mg^2+^. These conditions caused spontaneous epileptiform activities to arise from unpredictable foci (Demir et al., 1998). In the second model, slices were perfused with 0.5 mM Mg^2+^ and 100 μM 4-aminopyridine (4-AP) before receiving local N-methyl-D-aspartate (NMDA) applications which trigger a focal ictal-like discharge (Gomez-Gonzalo et al., 2010; Losi et al., 2010). This latter model offers the unique opportunity to repetitively evoke an ictal-like discharge from the same restricted site and it thus represents a powerful approach to analyze the contribution of different molecules and signaling pathways to the generation of epileptiform events. By using fast laser-scanning microscope Ca^2+^ imaging from neurons and astrocytes we monitored epileptiform network activities in these two models and found that local applications with both IL-1β and HMGB1 could rapidly lower the threshold for the initiation of focal ictal discharges.

## Materials and methods

### Brain slices and loading

All experimental procedures were authorized by the Italian Ministry of Health; all efforts were made to minimize the number of animal used and their suffering. Coronal cortical-hippocampal slices were obtained from 13 to 17 days old Wistar rats as previously described (Fellin et al., 2004). Briefly, brain was removed and put into ice-cold cutting solution containing (in mM): 120 NaCl, 3.2 KCl, 1 KH_2_PO_4_, 26 NaHCO_3_, 2 MgCl_2_, 1 CaCl_2_, 10 glucose, 2 Na-pyruvate, and 0.6 ascorbic acid at pH 7.4 (with 5% CO_2_/95% O_2_). Slices were obtained by cutting with a Leica Vibratome VT1000S (Mannheim, Germany) in the presence of the NMDA receptor inhibitor kynurenic acid (2 mM). Slices were recovered for 15 min at 37°C and then loaded with the Ca^2+^ sensitive dye Oregon Green 488 BAPTA-1 acetoxymethyl ester (OGB-1 AM, 20 μm; Invitrogen, Carlsbad, CA, U.S.A.) for 60 min at 37°C. Dye loading was performed in the cutting solution containing sulfinpyrazone (200 μM), pluronic acid (0.12%), and kynurenic acid (1 mM). After loading, slices were recovered and kept at room temperature in the presence of 200 μM sulfinpyrazone.

### Calcium imaging

Brain slices were continuosly perfused in a submerged chamber (Warner Instruments, Hamden, CT, USA) with a recording solution containing (in mM): 120 NaCl, 3.2 KCl, 1 KH_2_PO_4_, 26 NaHCO_3_, 1 MgCl_2_, 2 CaCl_2_, 10 glucose at pH 7.4 (with 5% CO_2_/95% O_2_) and Ca^2+^ signal images (512 × 512 pixels) were acquired by a TCS-SP5-RS confocal microscope (Leica Microsystem, Germany) equipped with a 20× water/objective (NA, 1.0) and a CCD camera for differential interference contrast. Time frame acquisitions from 314 to 491 ms (with 6–7 line averaging) were used. The Ca^2+^ responsiveness in neurons and astrocytes was determined on the basis of a threshold criterion. The onset was identified by the change in Δ F/F0 that should be more than two standard deviations over the average baseline and remained above this value in the successive frames for at least 2 s (two to six frames, depending on the frame acquisition rate). No background subtraction or other manipulations were applied to digitized Ca^2+^ signal images, with the exception of difference images in Figure 2 that were obtained by subtracting the pre-stimulation Ca^2+^ image from the post-stimulation Ca^2+^ image.

### Slices models of epileptic activity and IL-1β/HMGB1 applications

At a cellular level interictal and ictal-seizure like events were identified as intense and synchronous discharges that involve large neuronal population (Gomez-Gonzalo et al., 2010; Gómez-Gonzalo et al., 2011). In Ca^2+^ imaging of cortical slice the duration of the epileptic event was an important criterion for classifying interictal and ictal events. Interictal-like events lasted less than 3 s (D'Antuono et al., 2010), whereas ictal-like events were sustained for tens of seconds with a final pattern of highly synchronous activity that involved fundamentally all neurons in the recording field. In a first model, epileptiform activities were induced upon perfusion of cortical slice preparations with a recording solution containing the GABA_A_ receptor inhibitor picrotoxin (50 μM, Sigma-Aldrich, Milan, Italy) in the virtual absence of Mg^2+^. The ictal latency was evaluated by measuring the time between the onset of the picrotoxin perfusion and the first ictal-like event. In the second model, as previously described by Losi et al. (2010), focal ictal-like discharges were evoked by local NMDA applications in the presence of 4-aminopyridine (4-AP, 100 μM; Abcam, Cambridge, UK) and 0.5 mM MgCl_2_. A pressure ejection unit (PDES, NPI Electronics, Germany) was used to apply pressure pulses (4–10 psi, 200–600 ms duration) to a pipette containing 1 mM N-methyl-D-aspartate (NMDA, Sigma-Aldrich) localized on layers V-VI of EC. Pulse pressure (or duration) was increased until a double NMDA pulse evoked an ictal-like event while a single NMDA pulse induced only a transient local Ca^2+^ response. The parameters for successive stimulations remained unchanged over the entire recording experiment. The pipettes containing IL-1β (500 ng/ml rat recombinant IL-1β, Sigma-Aldrich) or HMGB1 (1 μM LPS-free HMGB1, HMGBiotech, Milan, Italy) were placed close to EC neurons and the inflammatory cytochines were locally applied by pressure pulses (2–5 psi for 200–600 ms) every 20 s for 15 min, just before picrotoxin or single NMDA pulses. Control experiments used the saline solution (1.2 M NaCl, 50 mM KCl, 10 mM NaH_2_PO_4_, 200 mM HEPES) in which the cytochines were dissolved.

A number of experiments were performed in the continuous presence of 2 μM tetrodotoxin (TTX, Abcam). In these experiments, repetitive single NMDA pulses (one every 2 min) were applied for 10 min before and 10 min after IL-1β, HMGB1 or saline pulse applications (one every 20 s) and both number of responsive neurons and amplitude of the Ca^2+^ response were evaluated. Groups of TTX experiments were preceded by ictal-like activity in 4-AP to test if the epileptic activity could change the effects of the cytochines on the NMDA-mediated Ca^2+^ response.

### Data analysis

Data analysis of Ca^2+^ signal was performed with LEICA LAS-AF (Leica), ORIGIN 7.5 (Microcal software, Northampton, MA, U.S.A.) and MATLAB (The MathWorks, Natick, MA, USA). Ca^2+^signal changes from regions of interest were measured by Δ F/F_0_, where F_0_ is the baseline fluorescence. In the picrotoxin experiments we evaluated different neuronal parameters (latency and duration of the first ictal-like discharge event, interictal and ictal-like event frequency). In this group of experiments we applied the unpaired Student's *t*-test and compared the cytokine treated groups with the control-saline treated group. In the 4-AP/NMDA experiments we evaluated the number of responding cells and their Ca^2+^ activity (maximal Δ F/F_0_ point) in response to a single NMDA pulse, both before and after cytokine applications. The Mann-Whitney non-parametric test on normalized values was used, with *p*-values ≤ 0.05 taken as statistically significant. Data are shown as mean ± standard error of the mean (S.E.M.).

## Results

### IL-1β and HMGB1 favor ictal-like discharge generation

#### Picrotoxin/low Mg^2+^ entorhinal cortex slice model

**Figure 1:**
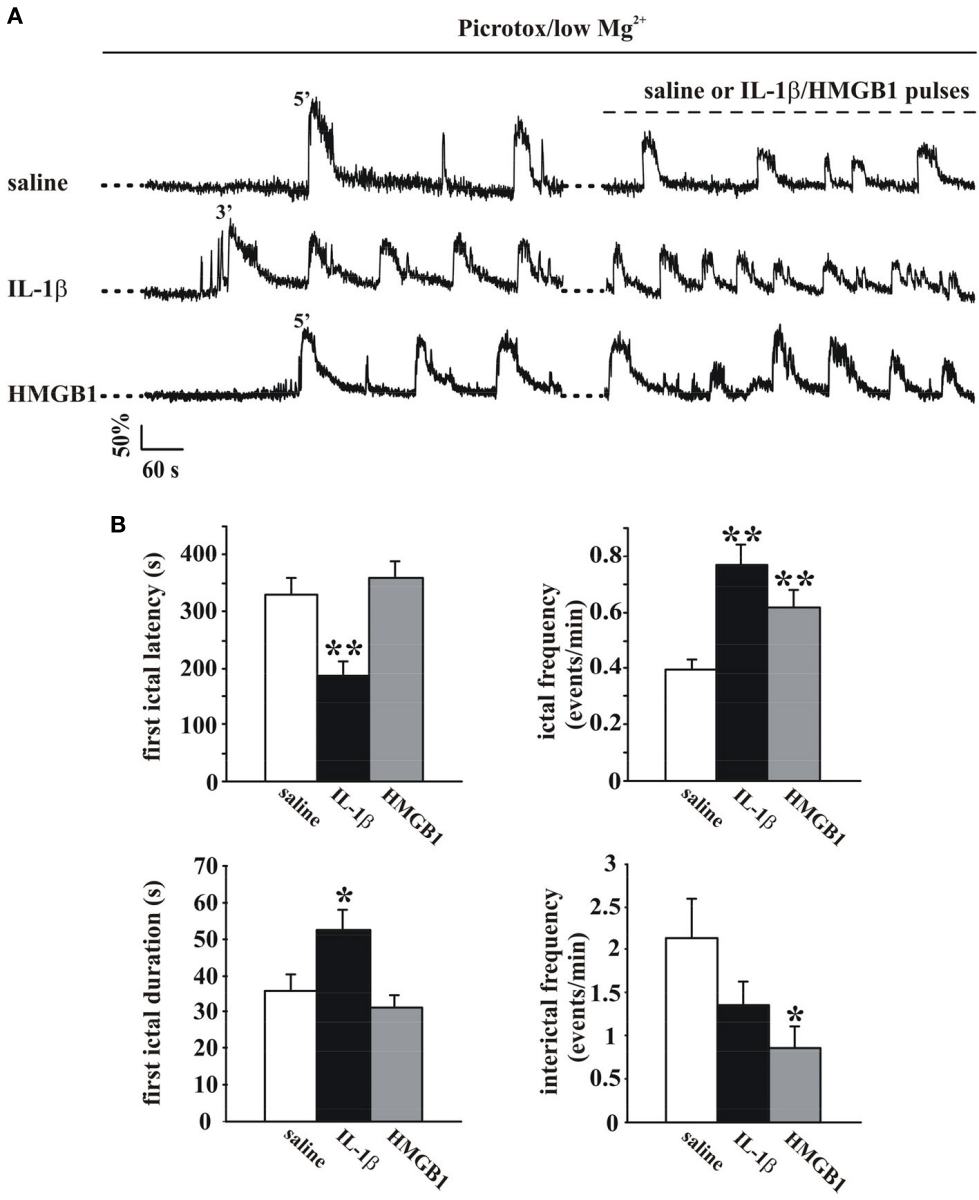
**IL-1β and HMGB1 applications favor ictal-like event generation in the low Mg^2+^/Picrotoxin model**. **(A)** Ca^2+^ changes in representative neurons from a rat EC slices perfused with low Mg^2+^/Picrotoxin in the absence (upper trace, saline) and in the presence of IL-1β or HMGB1 pulse applications (middle and lower traces, respectively). **(B)** Histograms representing the quantification of the mean latency and duration of the first ictal-like event (left panels) and ictal and interictal frequency (right panels) in saline-treated (white bars, 11 slices, 7 animals), IL1β-treated (black bars, 8 slices, 4 animals) and HMGB1 treated (gray bars, 9 slices, 5 animals) slices. Unpaired Student's *t*-test between saline and cytokine treated experiments, ^*^p = 0.05, ^**^p = 0.01.

The change in the cytosolic Ca^2+^ signal is a useful tool to study seizure, ictal-like discharges in neuronal ensembles since it reflects faithfully the action potential bursts that characterize the epileptic discharges in individual neurons (Gomez-Gonzalo et al., 2010; Losi et al., 2010). To start investigating a possible role of inflammatory agents, such as IL-1β and HMGB1, in ictogenesis we loaded EC slice preparations from young rats with the Ca^2+^ indicator OGB-1 and epileptiform activities were observed to arise spontaneosuly after a prolonged slice perfusion with the GABA_A_ receptor antagonist picrotoxin in low extracellular Mg^2+^. We found that with respect to the onset of the picrotoxin/low Mg^2+^ perfusion, the first ictal-like event occurred with a significantly shorter latency in slices pretreated with IL-1β pulses (one every 20 s for 15 min; see Materials and Methods; *p* = 0.0008, unpaired Student's *t*-test, *n* = 8) than in saline-pretreated slices (Figures 1A,B). Differently from IL-1β, HMGB1 affected neither the latency of the first ictal event nor the ictal-like duration (*n* = 9). However, both IL-1β and HMGB1 significantly increased the overall frequency of ictal-like events and reduced interictal events (Figure 1B).

#### Focal seizure model

**Figure 2:**
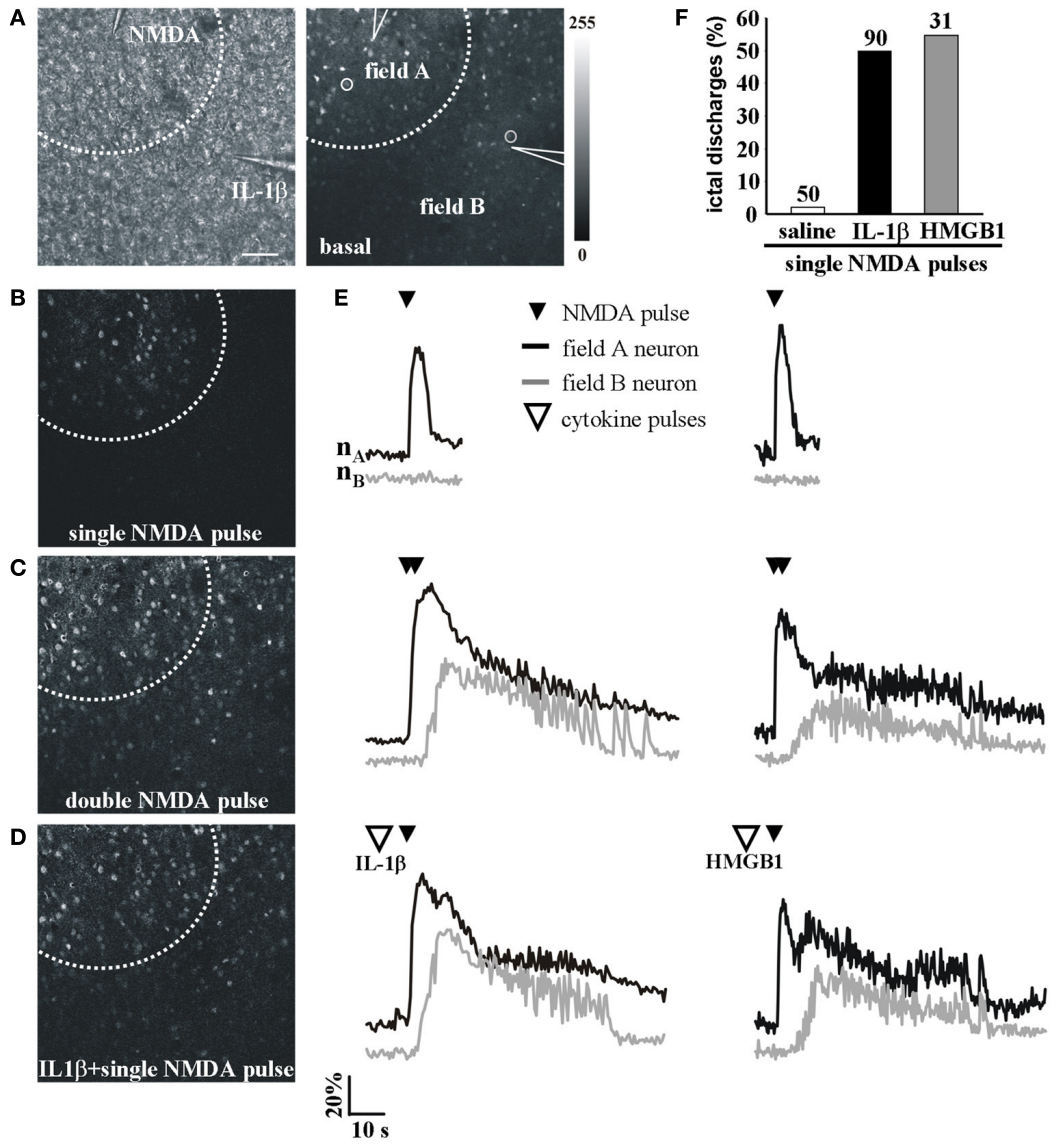
**IL-1β and HMGB1 local applications enhance generation of focal ictal-like events**. **(A)** DIC (left) and fluorescence (right) images of a cortical region from an EC slice showing the NMDA and the IL-1β pipettes. Scale bar 100 μ. **(B–D)** Difference images of the neuronal Ca^2+^ increase upon a single ineffective NMDA pulse **(B)**, a double **(C)** NMDA pulse that successfully evoked a focal ictal-like event, and **(D)** a single NMDA pulse that after IL-1β also evoked a focal event. **(E)** Ca^2+^ changes in representative neurons of field A (nA) and field B (nB) upon a single, a double NMDA pulses and a single NMDA pulse applied after IL-1β (left) or HMGB1 (right). **(F)** Quantitative evaluation of successful single NMDA pulses in saline-treated (50 pulses, 16 experiments, 11 animals) IL-1β (90 single NMDA pulses, 26 experiments, 18 animals) and HMGB1 (31 pulses, 10 experiments, 6 animals).

**Figure 3:**
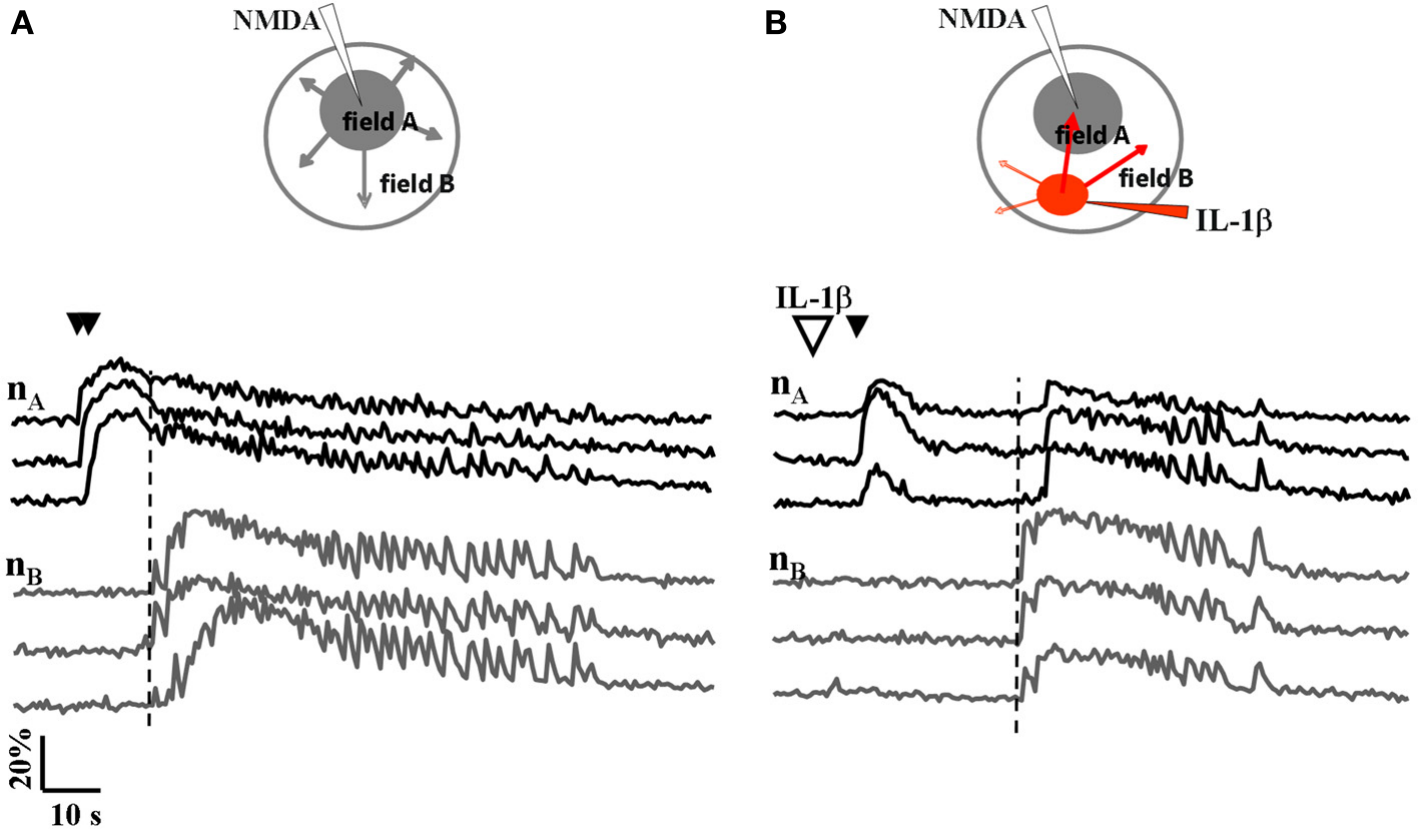
**A focal ictal-like event can initiate at the site of IL-1β local applications**. **(A)** Schematic view and neuronal Ca^2+^ changes during a focal ictal evoked by a double NMDA pulse (double black arrows). The focal ictal-like event arose in field A neurons (nA), close to the NMDA pipette, and then propagate to neurons in B (nB). **(B)** In the same slice, after IL-1β applications (red spot in the inset, and big white arrow), a focal ictal was evoked by a single NMDA pulse. The Ca^2+^ change that marked the onset of the focal ictal-like event occurred first in neurons from the site of IL-1β applications and then it propagated to neurons from the site of the single NMDA pulse with a delay of about 20 s. Similar results were observed in 4 out of 26 ictal-like discharges. The vertical dashed line marks the response in neurons from field B.

We next asked whether IL-1β and HMGB1 can also affect focal ictal generation. To this aim, we used an EC slice model in which focal ictal-like discharges were reproducibly generated at a restricted site by perfusing the slice with 100 μM 4-AP and 0.5 mM Mg^2+^, and stimulating a small number of neurons with pressure pulses applied to an NMDA-containing glass pipette. As previously reported (Gomez-Gonzalo et al., 2010; Losi et al., 2010), in this model slices treated with a double, but not a single NMDA pulse triggered a focal ictal-like event. The differential contrast image (DIC) and the fluorescence images in Figure 2A show a representative field in EC layer V-VI, the NMDA- and the IL-1β-containing pipettes. As illustrated by the difference images generated by subtracting the fluorescence image captured at basal conditions to that obtained after the NMDA stimulation (Figures 2B–D), a single NMDA pulse induced only a transient Ca^2+^ raise in a limited number of neurons close to the pipette tip, an area that we defined as the focal area (field A; Figure 2B). In contrast, a double NMDA pulse stimulation evoked a stronger activation of field A neurons as well as Ca^2+^ elevations in the surrounding neurons (field B) with the typical pattern of an ictal-like discharge (Figure 2E). The ictal event evoked by a double NMDA pulse was highly reproducible while only 1 out of 50 single NMDA pulse performed in 16 slices generated an ictal event within 45 min of 4-AP perfusion. We found that if a single subthreshold NMDA stimulation (that was in general ineffective) was preceded by IL-1β or HMGB1 applications, a focal ictal-like event was evoked in 45 of 90 and 17 of 31 single pulse stimulations, respectively, suggesting that the cytokines can lower the threshold for ictal generation (Figure 2F). In a few IL-1β experiments (4 out of 26), we also noted that an ictal-like event was not generated, as usually, at the site of NMDA applications, but rather at the site where IL-1β was applied (Figure 3). According to the Ca^2+^ signal change in these experiments, the focal ictal-like event initiated, indeed, in neurons from the IL-1β site and secondarily spread to neurons from the NMDA stimulation site (Figure 3B).

### After IL-1β and HMGB1 local applications neurons and astrocytes increase their response to NMDA

**Figure 4:**
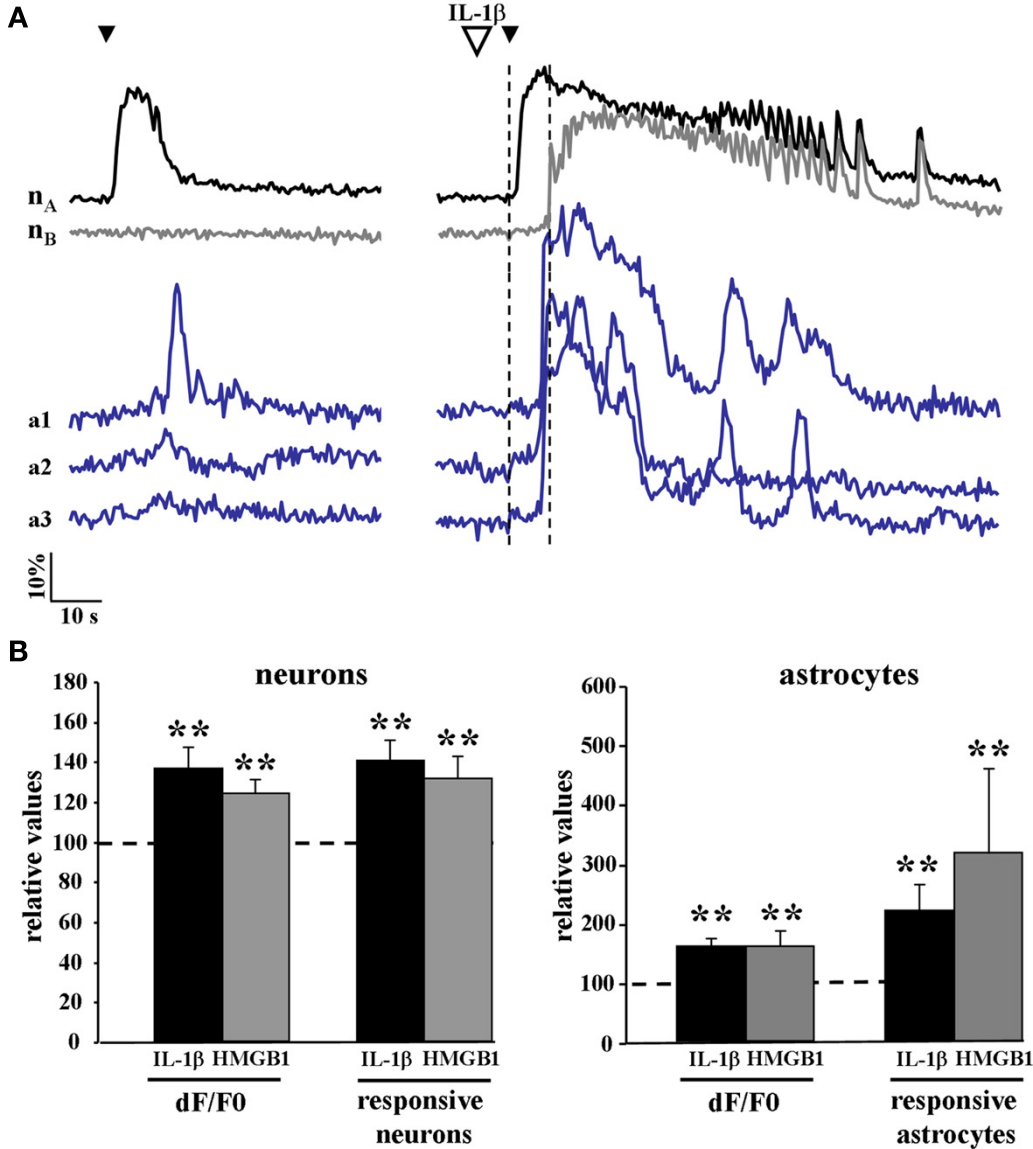
**The Ca^2+^ responsiveness in neurons and astrocytes to a single NMDA pulse was increased following IL-1β and HMGB1 applications**. **(A)** representative Ca^2+^ changes in neurons and astrocytes evoked by a single NMDA pulse in the absence (left traces) and presence (right traces) of IL1β. The vertical dashed lines indicate the time interval between the NMDA pulse and the Ca^2+^ rise in neurons surrounding the focus that marked the ictal discharge onset. **(B)** Bar histograms of neuron and astrocyte amplitude response to a single NMDA pulse applied after IL-1β (black bars, 12 slices, 614 neurons and 356 astrocytes, 11 animals) or HMGB1 (gray bars, 7 slices, 351 neurons and 154 astrocytes, 5 animals). Mann-Whitney test, ^**^p = 0.01.

We then asked whether cytokines could lower the threshold for the generation of focal ictal-like discharges by enhancing the response of the epileptogenic network to NMDA stimulation. We measured the number of activated neurons and astrocytes as well as the amplitude of the Ca^2+^ change in these cells in response to a single NMDA pulse that was preceded by either saline or IL-1β (or HMGB1) applications. Since in this latter case the single NMDA stimulation induced a focal ictal-like event, we restricted our analysis to the initial phase of the response to NMDA, i.e., the time interval between the NMDA pulse and the Ca^2+^ rise in neurons surrounding the focus that marked the ictal discharge onset (dashed vertical lines in Figure 4A). As reported in the bar histogram of Figure 4B, both Ca^2+^ elevation amplitude (Δ F/F0) and the number of neurons and astrocytes activated by a single NMDA pulse were significantly increased after IL-1β and HMGB1 applications.

### IL-1β and HMGB1 action depends on synaptic transmission

**Figure 5:**
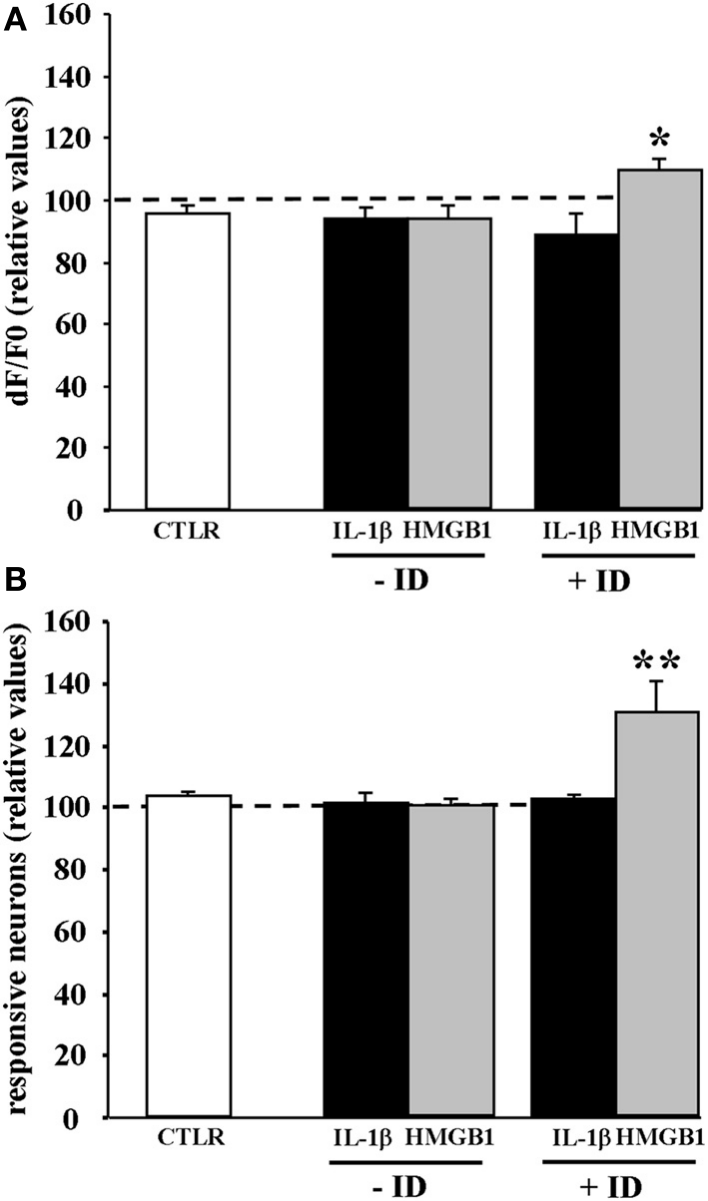
**HMGB1 can act directly onto NMDA receptors after the brain tissue experienced epileptiform activities**. Mean peak amplitude of the neuronal Ca^2+^ change **(A)** and mean number of responsive neurons **(B)** evoked by a single NMDA pulse applied in 0.5 mM Mg^2+^ and TTX. Slices were treated with IL1β or HMGB1 and experienced (+ID) or did not experience (−ID) an ictal-like discharge before TTX application. IL1β (black bars; 12 slices, 465 neurons, 9 animals), HMGB1 (gray bars; 13 slices, 575 neurons, 9 animals). The white bars correspond to data from control slices with saline applications instead of the cytokine applications (6 slices, 290 neurons, 3 animals). In each experiment Δ F/F0 max values and number of NMDA responsive neurons after cytokine treatment are normalized with respect to internal control. Neurons from the HMGB1 treated slices that experienced previous epileptic activity increase significantly their response to NMDA (7 slices, 5 animals). ^*^p = 0.05, ^**^p = 0.01, Mann-Whitney test.

IL-1β and HMGB1 can lower ictal threshold by enhancing either the direct response of neurons to NMDA or the synaptic transmission that follows NMDA receptor-mediated membrane depolarization. To clarify this issue, we performed experiments in the presence of 2 μM TTX that blocks synaptic transmission. In these experiments the amplitude of the Ca^2+^ change and the number of neurons activated by five successive single NMDA pulses (applied every 2 min) were measured before and after saline, IL-1β or HMGB1 pulses (applied every 20 s). We found that when synaptic transmission was blocked by TTX, both IL-1β and HMGB1 failed to enhance the NMDA-mediated Ca^2+^ response of neurons (*n* = 6 for both IL-1β and HMGB1 treated slices, Mann-Whitney test *p* = 0.37886 and 0.9362, respectively). This observation suggests that to lower the threshold of ictal-like discharges the two cytokines do not act directly on the NMDA receptor activation. However, if TTX was applied to a brain slice that had already experienced ictal-like discharges, HMGB1 (*n* = 7), but not IL-1β (*n* = 6), increased the responsiveness of neurons to a single NMDA pulse in terms of both of Ca^2+^ elevation amplitude (+11.7 ± 3,6%, *p* = 0.03 with Mann-Whitney test) and number of activated neurons (+30.2 ± 10%, *p* = 0.0021 with Mann-Whitney test) (Figure 5). These latter results raised the hypothesis HMGB1 can directly act on the NMDA receptor, but this event needs a sustained epileptic activity.

## Discussion

We here provide evidence that both IL-1β and HMGB1 can rapidly enhance the generation of epileptiform activities in two different EC slice models of focal ictal-like discharges. In the picrotoxin-low Mg^2+^ model, both agents applied locally to EC slices, increased the frequency of spontaneous ictal-like events, while in the 4-AP model of focally evoked ictal-like events they decreased ictal-like discharge threshold. In this latter model, Ca^2+^ imaging experiments revealed that the NMDA pulse applied to IL-1β- and HMGB1-treated slices evoked a larger activation of both neurons and astrocytes with respect to saline-treated slices. IL-1β or HMGB1 may lower ictal threshold by increasing the sensitivity of neurons to NMDA thus causing a larger recruitment of neurons into the initial episode of NMDA receptor-mediated excitation in a local circuit. These observations are compatible with the view that in a hyperexcitable brain network a focal seizure-like discharge can initiate when an episode of hyperactivity involves a critical mass of neurons.

Different observations in both experimental models and human TLE (for review, see Vezzani and Friedman, 2011) suggest an important role of inflammatory signals in epileptogenesis. The proconvulsant effect of IL-1β and HMGB1 was, indeed, previously reported in an *in vivo* mouse model in which IL-1β and HMGB1 enhanced seizure activity through a mechanism that involved the phosphorylation of the NMDA receptor subunit NR2B (Balosso et al., 2008). Other data obtained from cultured hippocampal neurons support the role of IL-1β in the modulation of NMDA channels through phosphorylation by Src kinases (Viviani et al., 2003). Our experiments in 4-AP/0.5 mM Mg^2+^ confirmed the capability of both cytokines to increase the responsiveness of neurons to NMDA receptor activation. However, after IL-1β applications in the presence of TTX, we could observe neither an increased amplitude of the neuronal Ca^2+^ response to NMDA nor an increased number of responsive neurons, suggesting an indirect IL-1β action on the NMDA receptor. While we can not exclude a contribution of IL-1β-induced phosphorylation in the proconvulsant effect of IL-1β, other mechanisms, such as activation of presynaptic transient receptor potential vanilloid channels (Rossi et al., 2012), may be also involved in the increased NMDA response.

Interestingly, we occasionally observed that after the initial response of neurons to the NMDA stimulation at the focus, an ictal-like event initiated at the site of IL-1β applications and then spread to other regions including the NMDA application site. This observation suggests that cells that had a direct contact with IL-1β may be more sensitive to NMDA receptor activation.

### HMGB1 enhances neuronal responsiveness to NMDA receptor only after the tissue have experienced repetitive ictal-like events

When synaptic transmission was blocked by TTX, both IL-1β and HMGB1 failed to enhance the NMDA-mediated Ca^2+^ response of neurons, suggesting that to lower the threshold of ictal-like events the two cytokines do not act directly on NMDA receptors. However, HMGB1, but not IL-1β, directly increased the Ca^2+^ response to NMDA applications only if the brain tissue experienced an epileptic event prior to NMDA challenges. Note that in the picrotoxin/low Mg^2+^ model HMGB1 significantly increased the mean frequency of ictal-like events, but, differently from IL-1β, it had no effect on the latency of the first epileptic event. We advance the hypothesis that epileptiform activity exerts a priming effect on the tissue to become sensitive to the HMGB1 action. Consistent with this hypothesis, an upregulation of TLR4 expression, i.e., the primary HMGB1 receptor, has been described in both neurons and astrocytes in human cortical malformations (Zurolo et al., 2011) as well as after seizures in animal models (Maroso et al., 2010). It should be noted that since the time scale of our experiments can hardly be consistent with a *de novo* synthesis of this receptor, an alternative mechanism may involve an accelerated mobilization of pre-synthesized receptor from the Golgi to the membrane (Saitoh and Miyake, 2009; McGettrick and O'Neill, 2010). Also to be considered is that rapid changes of the redox state of HMGB1, that may occur during epileptiform activities, are critical for NMDA receptor phosphorylation by this inflammatory agent (Balosso et al., 2014).

In a previous study, we showed that cultured astrocytes can release HMGB1 following IL-1β stimulation (Zurolo et al., 2011). Also noteworthy is that astrocytes, microglia and neurons (expressing TLR4) may respond to HMGB1 stimulation with a production of several pro-epileptogenic inflammatory mediators (Andersson et al., 2005; Kim et al., 2006; Pedrazzi et al., 2007) providing a positive feedback loop that can amplify neuronal excitability.

Since we are investigating the action of two inflammatory agents, on a cautionary note we have to consider the inflammatory components that are potentially induced by slice cutting procedures. It is known that microglia are quickly activated during these procedures and may contribute to the inflammatory status of the slices. Microglia may also actively participate in the modulation of excitatory neurotransmission by recruiting astrocytes via ATP release (Pascual et al., 2012). Nevertheless, our model can not be considered a model of neuroinflammation and this has to be taken into account in evaluating the action of IL-1β and HMGB1 that we reported here.

### Astrocyte calcium elevation

In an excitatory loop with neurons, astrocytes have been previously shown to promote neuronal synchronization in local circuits (Fellin et al., 2004) and through this action to enhance the generation of focal ictal-like events in EC slice preparations (Gomez-Gonzalo et al., 2010). A selective inhibition of Ca^2+^ signals in astrocytes increased focal ictal-like threshold, whereas a selective activation of Ca^2+^ increases in astrocytes enhanced ictal generation. In the present study, with respect to controls we observed a significant higher response of astrocytes to a single NMDA pulse applied in IL1β- or HMGB1-pretreated slices. Moreover, blocking synaptic transmission with TTX prevented Ca^2+^ elevations in astrocytes. This evidence suggests that the Ca^2+^ elevation in astrocytes depends on the synaptic activity and that the proconvulsant effects of IL1β and HMGB1 may reflect a regulation of the neuron-glia communication.

Astrocytes have been, indeed, shown to express a large variety of metabotropic and ionotropic glutamate receptors (Schipke et al., 2001; Lalo et al., 2006; Verkhratsky and Kirchhoff, 2007; D'Antoni et al., 2008; Lundborg et al., 2011). Furthermore, an increasing number of studies provide evidence that the release of gliotransmitters, such as glutamate, ATP or D-serine, can modulate basal synaptic transmission (Di Castro et al., 2011; Panatier et al., 2011) and short- and long-term changes of synaptic strenght in both *in vitro* (Pascual et al., 2005; Serrano et al., 2006; Henneberger et al., 2010; Navarrete and Araque, 2010; Min and Nevian, 2012) and *in vivo* models (Takata et al., 2011; Navarrete et al., 2012; Chen et al., 2013).

By secreting and sensing a large variety of cytokines and chemokines astrocytes may provide a fundamental contribution in the control of the inflammatory status of the brain and through this mechanism to contribute to the generation of epileptiform activities (Aronica et al., 2012). Here we propose that the proconvulsant action of the inflammatory molecules IL-1β and HMGB1 involves also an amplification of neuron-astrocyte reciprocal signaling in local circuits which favors neuronal synchronization and ultimately leads to a decreased threshold for focal ictal-like events.

In conclusion, although the precise underlying cellular mechanism needs to be investigated, our findings demonstrate that both IL-1β and HMGB1 can rapidly affect neuronal excitability and under proepileptic conditions lower the threshold of focal ictal-like discharges. Our findings raise the possibility that targeting these inflammatory pathways may represent an effective therapeutic strategy to prevent seizures.

### Conflict of interest statement

The authors declare that the research was conducted in the absence of any commercial or financial relationships that could be construed as a potential conflict of interest.
